# Metacognitive training and AI-based Socratic scaffolding in pre-service teachers’ reflective practice: a dual-study experimental design

**DOI:** 10.3389/fpsyg.2026.1960962

**Published:** 2026-10-13

**Authors:** Wen Li Zhang, Sijie Sun, Lifan Hu, Li Liu, Wenwuyu Gao, Shuangqi Li

**Affiliations:** 1 Normal College, Changzhou Institute of Technology, Changzhou, China; 2 Department of Public Education, Anhui Finance & Trade Vocational College, Hefei, China; 3 College of Preschool Education, Shanghai Normal University Tianhua College, Shanghai, China

**Keywords:** AI empowerment, human-computer interaction, interaction effect, metacognitive training, momentary effect, reflective practice

## Abstract

This study examined whether metacognitive strategy training strengthens the contribution of an AI-based Socratic reflection system to pre-service teachers’ reflective practice. Study 1 used a 2 (metacognitive training: present vs. absent) × 2 (tool condition: AI-based Socratic scaffolding vs. traditional digital tools) randomized between-subjects design (*N* = 60; *n* = 15 per cell). Written reflection quality at post-test was analyzed by ANCOVA with pre-test performance as the covariate. The metacognitive-training main effect was significant, *F*(1, 55) = 33.89, *p* < 0.001, *η*^2^*p* = 0.381, as was the tool-condition main effect, *F*(1, 55) = 9.63, *p* = 0.003, *η*^2^*p* = 0.149. More importantly, the metacognition × tool interaction was significant, *F*(1, 55) = 10.91, *p* = 0.002, *η*^2^*p* = 0.165. Under metacognitive training, AI-based Socratic scaffolding produced a significant model-estimated advantage over traditional digital tools (Δ*M* = 0.56, *p* < 0.001), whereas the tool difference was not significant without metacognitive training (Δ*M* = −0.03, *p* = 0.821). Study 2 examined dialogue logs from the two AI conditions. Participants who received metacognitive training generated a substantially larger proportion of higher-order prompts than participants who received placebo training (44.1% vs. 12.2%), t(28) = 7.14, *p* < 0.001, *d* = 2.61. These process data are consistent with the proposed account that metacognitive preparation changes how learners engage with AI, although they do not constitute a formal mediation test. The findings suggest that, within this configured Socratic AI environment, metacognitive preparation is an important condition for realizing higher-quality reflective practice.

## 1 Introduction

### 1.1 The Core status of reflective practice in the professional development of pre-service teachers

The accelerating integration of artificial intelligence (AI) into education compels educators to confront a pivotal question: will AI serve merely as a convenient tool that reinforces surface-level cognition, or can it become an empowering instrument that catalyzes deep professional reflection? This question anchors the present inquiry.

Reflective practice is deeply rooted in educational theory. John Dewey first introduced reflection into the educational domain, establishing its philosophical foundation by asserting that everyday experience, when critically examined, is transformed into meaningful learning (Dewey, 1938). Extending this intellectual heritage, Donald Schön innovatively applied reflection theory to professional practice, proposing two interconnected modes of reflection, namely reflection-in-action (immediate diagnosis and dynamic adjustment) and reflection-on-action (retrospective reconstruction and conceptualization), which collectively enable practitioners to move beyond the constraints of “technical rationality” and evolve from passive recipients of theory into active inquirers and problem-solvers (Kinsella, 2007; Schön, 1983, 1987). Through recursive cycles that interconnect theory and practice, problems and insights, actions and feedback within authentic, complex teaching contexts, teachers cultivate contextualized and transferable practical knowledge, gradually construct their professional identity, and consequently develop both intrinsic motivation and a robust sense of self-efficacy that sustains lifelong professional growth (Wang and Dou, 2013; Wei, 2020; York-Barr et al., 2005).

Nevertheless, the reflective practice of pre-service teachers in China currently confronts a threefold predicament. First, reflection tends toward superficiality, frequently remaining confined to documenting instructional techniques rather than interrogating the fundamental nature of teaching (Wang, 2024). Second, reflection is often decoupled from authentic teaching contexts and concrete behaviors, devolving into a formalized exercise of textual production (Chen, 2022). Third, reflection is constrained by rigid formats and characterized by delayed feedback, impeding the formation of timely and actionable pedagogical improvements. Schön's (1983) evocative metaphor of the “swampy lowlands of professional practice” vividly captures the complexity, uncertainty, and value conflicts that teachers navigate in real-world classrooms. It is precisely within these swampy, dilemma-ridden terrains that authentic reflective practice, the capacity for spontaneous adaptation and the extraction of essential insights from unique situations, becomes most indispensable. In this context, the emergence of AI tools casts both new possibilities and new perils upon this perennial educational challenge. Can AI serve as a compass through these lowlands, enabling pre-service teachers to accumulate reflection data more efficiently, excavate tacit knowledge more deeply, and structure their reflective frameworks more systematically? Or will it lure teachers onto a deceptively efficient “high ground,” ensnaring them in the comfort of algorithmic recommendations and thereby foreclosing the very possibility of deep reflection? This is the core question that the present study directly and systematically addresses (Figure 1).

**Figure 1:**
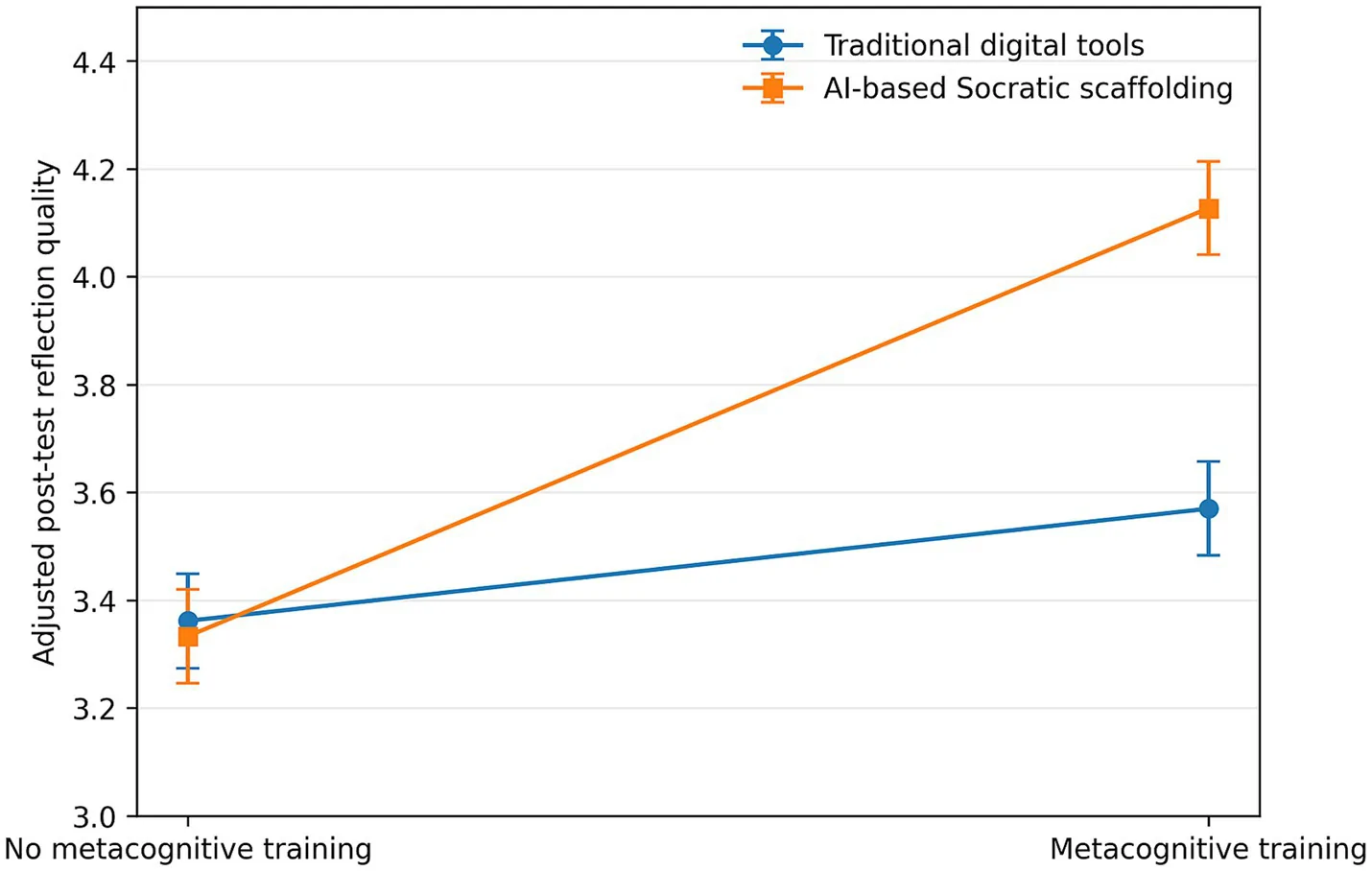
Model-estimated interaction pattern for written reflection quality. Error bars represent standard errors.

### 1.2 The dual role of generative AI in cognitive tasks (opportunities and challenges in higher education)

Generative AI tools are rapidly permeating higher education, emerging as a transformative force that is reshaping the pedagogical landscape. A growing body of research converges on the view that AI chatbots, particularly large language models such as ChatGPT, hold considerable promise for applications across diverse educational contexts (Crompton and Burke, 2023). Positioned as “cognitive empowerment tools,” these technologies are fundamentally altering patterns of teacher-student interaction and instructional practices. University students have broadly adopted AI tools to support a wide spectrum of academic activities, including text summarization, information extraction, content rewriting, thesis writing assistance, cross-lingual translation, and grammatical editing, all directed toward enhancing academic efficiency and output quality (Bearman et al., 2023; Bates et al., 2020; Chan and Hu, 2023). Students consistently perceive AI chatbots as offering substantial advantages, such as instantaneous feedback, personalized learning support, and reduced cognitive load, which in turn foster learning motivation and self-efficacy (Almufarreh, 2024; Delcker et al., 2024; Fošner, 2024; Grájeda et al., 2024).

Nevertheless, these opportunities are accompanied by considerable risks. First, students maintain a cautious stance regarding the accuracy and reliability of AI-generated content, expressing concern that the “hallucination” phenomenon inherent in large language models may introduce biased or erroneous information into their learning processes (Ansari et al., 2023). Second, overdependence on AI tools raises serious concerns about academic integrity, particularly the exacerbation of ghostwriting practices, and may undermine student agency and intellectual autonomy (Bearman and Ajjawi, 2023). Most critically, excessive reliance on generative AI has been shown to negatively affect higher-order cognitive capacities, including critical thinking, reflective learning, and creative reasoning, which constitute the core of deep learning. This may ultimately culminate in cognitive offloading, whereby students externalize cognitive labor to AI systems at the expense of their own intellectual development (Baidoo-Anu and Ansah, 2023; Kasnakoglu, 2023).

### 1.3 Metacognition as the key to unlocking AI’S potential

Potential Metacognition, broadly defined as an individual’s awareness, monitoring, planning, and regulation of their own cognitive processes, serves as a critical mechanism that enables learners to achieve autonomous regulation when confronting complex tasks. Flavell's (1979) seminal work first established the foundational status of metacognition in learning theory, while Zimmerman (2002) subsequently articulated its intrinsic interconnection with self-regulated learning (SRL). A robust body of empirical evidence indicates that well-designed heuristic questioning and reflective tools not only significantly enhance students’ metacognitive abilities (Schraw and Dennison, 1994; Desoete, 2008) but also facilitate their self-regulation throughout the learning process.

Crucially, effective utilization of AI differs fundamentally from interaction with traditional learning tools. It transcends mere mastery of operational procedures and instead constitutes a higher-order cognitive competency that encompasses goal setting, process monitoring, and outcome evaluation. The theoretical essence of this competency lies in the innovative application and extension of SRL theory (Zimmerman, 2002) to the specific context of human-computer interaction. Proficient AI users systematically deploy a three-tier metacognitive strategy, comprising pre-planning (establishing interaction objectives and designing prompt architectures), process monitoring (evaluating the quality of AI feedback and dynamically adjusting prompting strategies), and post-evaluation (reviewing interaction outputs and reflecting on cognitive gains). Through this recursive cycle, they transform AI from a passive text-generation apparatus into a flexible and responsive cognitive partner capable of providing adaptive scaffolding (Collins et al., 1989). It is precisely this iteratively self-regulative process, driven by deliberate metacognitive control, that unlocks and amplifies AI’s empowering potential for supporting deep reflective practice. Prior research has corroborated that metacognitive strategy instruction significantly improves learners’ interaction efficacy with novel technologies (Kramarski and Michalsky, 2010). On the basis of this theoretical reasoning, we posit that systematic metacognitive strategy interventions, including structured metacognitive training and reflective prompt tool design (Schraw, 1998), can substantially enhance users’ self-regulatory capacities and learning motivation, thereby magnifying the empowering effect of AI on their reflective practice.

### 1.4 Research gap, purpose, and research questions

Research on generative AI in higher education has expanded rapidly, yet much of the evidence remains descriptive or correlational, emphasizing adoption, perceived usefulness, and broad learning outcomes. Two issues remain insufficiently resolved in the context of teacher reflection. First, the learner-side cognitive preparation required for productive AI use is rarely separated experimentally from the technological support itself. Second, outcome-oriented studies seldom connect final reflective products with learners’ real-time interaction behavior.

The present research addresses these issues through two linked studies. Study 1 separates metacognitive strategy training from a specifically configured AI-based Socratic reflection environment in a randomized 2 × 2 factorial experiment. The tool condition should not be interpreted as generic access to an AI model: it combines generative capability with progressive probing, dialogic interaction, and adaptive feedback. Study 2 focuses on the two AI groups and analyzes their prompt behavior to determine whether metacognitive preparation is reflected in different forms of human-AI interaction.

The dual-study design therefore connects outcome-level experimental evidence with process-level behavioral evidence. It does not assume that Study 2 statistically mediates Study 1. A formal mediation claim would require a participant-level indirect-effect model linking training, prompt characteristics, and reflective outcomes. Accordingly, Study 2 is treated as process evidence consistent with the proposed mechanism rather than as a definitive mediation test.

RQ1 (Cumulative outcome): Do metacognitive strategy training and AI-based Socratic scaffolding interact in predicting pre-service teachers’ written reflection quality after baseline performance is taken into account?

RQ2 (Momentary process): Among users of the AI-based Socratic system, does metacognitive training correspond to a higher proportion of strategically complex and higher-order reflective prompts?

## 2 Literature review and theoretical framework

### 2.1 Conceptualizing reflective practice

In the field of teacher education, reflective practice is conceptualized as a multidimensional process of professional development, the core of which resides in achieving continuous pedagogical improvement through critical examination of teaching experiences. Van Manen's (1977) hierarchical model of reflection provides an essential framework for understanding this process, delineating three progressive levels: technical reflection, which concentrates on optimizing the effectiveness of instructional strategies; practical reflection, which interrogates the value presuppositions embedded in teaching behaviors; and critical reflection, which addresses the broader social, political, and ethical dimensions of educational practice (Hatton and Smith, 1995). Schön's (1983) complementary framework of reflection-in-action and reflection-on-action further enriches this theoretical architecture by emphasizing that reflection operates simultaneously as a real-time cognitive activity situated within the teaching episode and as a retrospective, deliberative review process. The organic integration of these two temporal dimensions positions reflective practice as a vital engine of teacher professional growth.

In recent decades, reflective practice theory has continued to evolve and deepen (Rolfe et al., 2001). The ALACT model proposed by Korthagen and Kessels (1999) operationalizes the reflection process as a dynamic cyclical system comprising five phases: Action, Looking back, Awareness of essential aspects, Creating alternative methods, and Trial. This model foregrounds the iterative and adaptive nature of reflective practice, underscoring the centrality of continuous self-assessment and flexible adjustment to sustained professional development.

Given the escalating complexity of contemporary teaching contexts, particularly in the face of digital pedagogical transformation, the depth and breadth of reflective practice have assumed even greater significance for teacher professional development. Reflection enables teachers to move beyond instrumental considerations of “how to teach” toward more fundamental inquiries into “why to teach this way” and “how digital technology fundamentally reshapes the nature of teaching.” Such reflective capacity is especially critical for the integrated cultivation of teachers’ Pedagogical Content Knowledge (PCK), Technological Knowledge (TK), and Technological Pedagogical Content Knowledge (TPACK), which collectively constitute the knowledge base for effective technology-enhanced teaching.

### 2.2 AI as a potential scaffolding tool for reflection

Within the field of learning technology research, AI is increasingly conceptualized as a scaffolding mechanism to support students’ reflective practice. However, drawing on human-computer collaborative design theory, this potential must be carefully weighed against its attendant risks. According to interaction design theory, effective human-computer collaboration should reduce cognitive load while preserving user agency (Norman, 1988). Sweller’s Cognitive Load Theory (CLT), however, introduces a more complex picture when applied to AI-enhanced learning environments (Shearer et al., 2021). Poorly designed AI interventions may paradoxically generate a “cognitive load paradox,” wherein pre-service teachers are forced to simultaneously manage three distinct cognitive burdens: (1) comprehending and internalizing AI-generated feedback, (2) executing the core reflective task, and (3) reconciling the cognitive tension between these two processes. More specifically, when AI-generated reflection prompts are excessively complex or misaligned with students’ existing mental models, they are likely to induce extraneous cognitive load, thereby undermining the depth and transferability of reflective outcomes. This reveals a critical theoretical-practical tension: not all AI deployment effectively reduces cognitive load; rather, the determining factor lies in whether the design adheres to cognitive science principles and whether the collaborative architecture genuinely embodies human-AI complementarity (Lodge et al., 2024).

In response to these dilemmas, the scholarly community has advanced the “Principle of Minimal Difference,” which posits that the gap between AI feedback and students’ mental models should be calibrated to remain within the Zone of Proximal Development (Vygotsky, 1978; Azevedo and Hadwin, 2005), thereby preventing cognitive offloading that may result from excessive challenge. To move beyond this impasse, the present study introduces the concept of “Cognitive Partnership.” In contrast to viewing AI either as an active transmitter of knowledge or a passive provider of information, this conceptualization emphasizes human-AI collaboration predicated on complementary cognitive strengths. Within this partnership, humans retain cognitive primacy and decision-making authority, while AI progressively internalizes students’ reflective processes through precisely calibrated, hierarchically structured scaffolding (Lodge et al., 2024; Overholser, 1997). This framework aligns with contemporary developments in Self-Regulated Learning Theory and scaffolding theory (Reiser and Tabak, 2014). Under this paradigm, ideal human-AI collaboration is characterized by four key attributes: human agency, AI assistance, dynamic adaptability, and transparency with explainability.

### 2.3 Metacognition as the engine of deep reflection

Metacognition, classically defined as “cognition about cognition” (Flavell, 1979), constitutes the cornerstone of self-regulated learning, encompassing both an individual’s knowledge of and active regulation over their own cognitive processes (Dimmitt and McCormick, 2012). Its regulatory function is widely conceptualized as a dynamic cyclical process comprising three interrelated phases: planning, monitoring, and evaluation (Schraw and Moshman, 1995). Planning involves establishing goals and selecting strategies prior to task engagement; monitoring entails real-time tracking of comprehension and progress during task execution; and evaluation encompasses critical reflection on outcomes and strategy effectiveness following task completion. These three phases do not operate as a fixed linear sequence but rather constitute a continuous, recursive feedback loop, wherein evaluation outcomes recursively inform subsequent planning. The efficacy of interventions designed to target these metacognitive components in enhancing academic achievement has been robustly confirmed by meta-analytic evidence (Ohtani and Hisasaka, 2018). At its core, metacognition repositions learners from passive recipients of information to active, strategic architects of their own knowledge construction.

Metacognition assumes particular significance in the context of open-ended, ill-structured tasks such as reflective practice, which lack predetermined solutions and depend heavily on learners’ sustained self-regulation. In such environments, metacognition functions as the central self-regulatory mechanism that steers cognitive processing through uncertainty. It enables individuals to strategically detect knowledge gaps, employ reasoning and analogy to bridge those gaps, and judiciously abandon unproductive lines of inquiry, all of which are essential for attaining deep understanding and creative problem-solving (Bransford et al., 2000). Moreover, metacognition provides a critical motivational maintenance function for coping with high-cognitive-load demands. By heightening individuals’ awareness of their own learning progress, it facilitates the transformation of cognitive difficulties into productive learning experiences, thereby sustaining the resilience and perseverance necessary for sustained reflection (Zimmerman, 2002). The contribution of metacognition to enhanced learning performance has been extensively validated across diverse educational contexts. Within the Cognitive Load Theory framework, Efklides (2008) advanced the construct of “affective metacognition,” foregrounding the profound interconnection between metacognitive and affective dimensions, a relationship that becomes especially salient in high-cognitive-load tasks. In the specific domain of reflective learning, international scholarship consistently indicates that cultivating students’ metacognitive abilities directly enhances the depth, engagement, and critical rigor of their reflective practice (Moon, 2013). Furthermore, metacognition operates synergistically with learner characteristics such as self-efficacy and learning motivation to further optimize reflective outcomes. Taken together, metacognition is not merely an ancillary meta-skill but rather functions as a core engine driving deep reflection, innovative learning, and complex problem-solving (Ma et al., 2022). Its underlying mechanisms merit continued investigation and pedagogical application.

### 2.4 Hypotheses

Grounded in Self-Regulated Learning Theory, Cognitive Load Theory, and metacognitive regulation research, the hypotheses distinguish learner preparation from the configured technological support. Because the experimental AI condition includes Socratic probing and adaptive dialogue, the hypotheses refer to AI-based Socratic scaffolding rather than to generic AI availability.

H1 (Interaction effect). Metacognitive training and tool condition will interact in predicting post-test written reflection quality, such that the benefit associated with AI-based Socratic scaffolding will be greater when participants have received metacognitive strategy training.

H1a. Within the metacognitive-training condition, participants using AI-based Socratic scaffolding will show higher post-test reflection quality than participants using traditional digital tools.

H1b. Within the placebo-training condition, the difference between AI-based Socratic scaffolding and traditional digital tools will be substantially smaller and will not reach statistical significance.

H2 (Process-level hypothesis). Within the AI-based Socratic condition, metacognitively trained participants will produce a higher proportion of prompts characterized by hypothesis generation, strategic evaluation, perspective taking, evidence checking, and other higher-order reflective features than placebo-trained participants.

## 3 Study 1: cumulative outcome experiment

### 3.1 Method

#### 3.1.1 Participants and design

Study 1 employed a 2 (Metacognitive training: present vs. absent) × 2 (Tool condition: AI-based Socratic scaffolding vs. traditional digital tools) randomized between-subjects factorial design with pre-test and post-test measurement. The primary outcome was written reflection quality assessed by trained raters. Sixty third-year pre-service teachers completed the full procedure and were distributed evenly across the four cells (*n* = 15 per condition).

The sample-planning rationale targeted the factorial interaction. Because the final cell size remained modest, a sensitivity interpretation was added to avoid overstating precision. With *N* = 60, *α* = 0.05, and approximately 55 residual degrees of freedom, the design is best suited to detecting moderate-to-large interaction effects. Small interaction effects may remain unstable, and all subgroup estimates should therefore be interpreted with corresponding caution.

Participants were recruited through online and on-campus advertisements. Eligibility required current enrollment in a teacher education program, basic information-technology skills, and voluntary participation. Ethical approval was obtained from the Humanities and Social Sciences Research Ethics Committee of Changzhou Institute of Technology. Written informed consent was obtained before stratified random allocation (Tables 1–9).

**Table 1:** Random assignment of participants.

Experimental condition	Tool condition	Metacognitive training	*n*
Group A	AI-based Socratic scaffolding	Present	15
Group B	AI-based Socratic scaffolding	Absent/placebo	15
Group C	Traditional digital tools	Present	15
Group D	Traditional digital tools	Absent/placebo	15

**Table 2:** Reliability and exploratory structural checks for the 16-item RPQ.

Stage	Overall *α*	Habitual action *α*	Understanding *α*	Reflection *α*	Critical reflection *α*	KMO	4-component variance
Pre-test	0.808	0.818	0.821	0.874	0.855	0.730	70.59%
Post-test	0.878	0.775	0.801	0.867	0.865	0.810	67.95%

Bartlett tests were significant at both time points (*p* < 0.001). The exploratory four-component check is reported cautiously because *N* = 60 is modest for psychometric validation.

**Table 3:** ANCOVA diagnostic and robustness checks.

Check	Statistic	*p*	Interpretation
Homogeneity of regression slopes	*F* = 1.50	0.225	No evidence of slope heterogeneity
Levene homogeneity of variance	*F* = 0.92	0.437	Variance assumption acceptable
Shapiro–Wilk residual normality	*W* = 0.985	0.668	Residual normality acceptable
Cook’s D sensitivity	Interaction *F* = 10.41	0.002	Interaction remains significant
HC3 robust inference	Interaction term	0.004	Interaction remains significant
Per-protocol sensitivity (*N* = 53)	Interaction *F* = 11.34	0.002	Interaction remains significant

**Table 4:** Reflection quality by experimental condition.

Condition	*n*	Pre-test *M* (SD)	Post-test *M* (SD)	Estimated M	SE
Metacognition + AI	15	3.12 (0.32)	4.14 (0.39)	4.13	0.09
Placebo + AI	15	3.02 (0.31)	3.31 (0.30)	3.33	0.09
Metacognition + traditional	15	3.03 (0.31)	3.55 (0.26)	3.57	0.09
Placebo + traditional	15	3.20 (0.27)	3.39 (0.40)	3.36	0.09

Estimated marginal means are calculated at the grand mean of pre-test reflection quality.

**Table 5:** ANCOVA for post-test written reflection quality.

Source	SS	df	MS	*F*	*p*	*η* ^2^ *p*
Pre-test	0.422	1	0.422	3.76	0.058	0.064
Metacognitive training	3.804	1	3.804	33.89	< 0.001	0.381
Tool condition	1.081	1	1.081	9.63	0.003	0.149
Metacognition × tool	1.224	1	1.224	10.91	0.002	0.165
Error	6.174	55	0.112	–	–	–

**Table 6:** Simple effects from the primary ANCOVA.

Comparison	Estimated difference	SE	t(55)	*p*
AI vs. traditional | metacognitive training	0.557	0.123	4.53	<0.001
AI vs. traditional | placebo training	−0.028	0.125	−0.23	0.821
Metacognitive vs. placebo | AI condition	0.794	0.123	6.44	< 0.001
Metacognitive vs. placebo | traditional condition	0.208	0.125	1.67	0.101

**Table 7:** Dimension-level post-test scores and interaction effects.

Dimension	*A*	*B*	*C*	*D*	*F* interaction	*p*	*η* ^2^ *p*
Description and evidence	3.90	3.38	3.57	3.47	3.36	0.072	0.058
Depth and attribution	4.24	3.32	3.46	3.39	22.84	<0.001	0.293
Theory and perspective taking	4.18	3.22	3.55	3.35	20.47	<0.001	0.271
Strategy generation and transfer	4.23	3.32	3.57	3.35	12.01	0.001	0.179
Structure and clarity	4.01	3.32	3.77	3.39	1.68	0.201	0.030

**Table 8:** Frequency and percentage distribution of cognitive levels in user prompts across groups.

Cognitive level	Metacognitive training + AI (*n* = 254 prompts)	Placebo + AI (*n* = 233 prompts)
Category 1: Lower-order cognition	54 (21.3%)	127 (54.5%)
Category 2: Intermediate cognition	88 (34.6%)	78 (33.3%)
Category 3: Higher-order cognition	112 (44.1%)	28 (12.2%)

**Table 9:** Illustrative interaction log patterns.

Group	Illustrative prompt pattern	Behavioral characterization
Placebo + AI	Is this approach right? Give me three reasons. / How should a teacher handle this? Help me write two improvement plans.	Fact-finding and answer-seeking; requests external judgments and ready-made solutions.
Metacognitive training + AI	Could this inconsistency between words and actions reflect technical rationality or a desire for classroom control? / If I used accommodation rather than correction, what opposing outcomes might occur?	Hypothesis testing, counterfactual reasoning, perspective taking, and evaluation of one’s own explanatory framework.

#### 3.1.2 Materials and experimental conditions

Two matched teaching-case vignettes were used as the pre-test and post-test tasks. The cases were designed to be comparable in narrative length, cognitive complexity, structural features, and pedagogical ambiguity. Participants had 30 min to analyze the case, identify key teaching problems, examine possible causes, propose improvement strategies, and justify their proposals.

The metacognitive intervention consisted of a 20-min module on planning, self-questioning, monitoring, and evaluation in teaching reflection. The placebo condition received a time-matched module on PowerPoint design and presentation skills. The placebo content was unrelated to reflective practice and served to control exposure to a structured training activity.

Participants assigned to the AI condition used a DeepSeek V3 web interface configured as a Socratic reflection partner. The system was designed to avoid immediately supplying ready-made solutions, to pose progressively probing questions, and to adapt follow-up questions to the user’s preceding response. The comparison condition used a conventional online text editor and the university’s digital repository without adaptive recommendations or dialogic guidance. The tool factor therefore bundles generative AI with adaptive Socratic scaffolding. The observed effect should not be generalized to unrestricted AI use or interpreted as a pure technology effect.

#### 3.1.3 Measures

Written Reflection Quality Rubric. Written reflection quality was scored with a five-dimension rubric derived from established reflection frameworks (Sparks-Langer et al., 1990; Ward and McCotter, 2004). The dimensions were Description of Teaching Facts and Evidence Awareness (20%), Depth of Reflection and Attribution Analysis (30%), Theory Connection and Perspective Taking (20%), Generation and Transfer of Improvement Strategies (20%), and Report Structure and Clarity (10%). Two trained raters scored all reports independently. The previously established ICCs (0.891 at pre-test and 0.903 at post-test) demonstrate strong scoring consistency. To distinguish reliability from validity, the revised manuscript also specifies the rubric’s theory-based content mapping, dimension weights, behavioral anchors, coder training procedure, and the use of matched cases. Inter-rater reliability is therefore treated as evidence of scoring consistency rather than as proof of construct validity by itself.

Reflective Practice Questionnaire (RPQ). The contextualized RPQ contained the 16 intended items from Kember et al., (2000) four-dimensional structure: habitual action, understanding, reflection, and critical reflection. Two background items administered before the RPQ were removed from RPQ scoring, correcting the earlier ambiguity in the exported total-score field. In the restructured 16-item scoring, internal consistency was acceptable to good. Overall Cronbach’s *α* was 0.808 at pre-test and 0.878 at post-test; subscale *α* values ranged from 0.818 to 0.874 at pre-test and from 0.775 to 0.867 at post-test. KMO values were 0.730 and 0.810, respectively, and Bartlett’s tests were significant at both time points (*p* < 0.001). A four-component exploratory check accounted for 70.59% of variance at pre-test and 67.95% at post-test, with rotated primary loadings ranging approximately from 0.60 to 0.90. Given the modest *N*, this factor check is treated as supportive rather than definitive evidence of measurement structure.

As an additional convergent check, post-test written reflection quality correlated moderately with the RPQ dimensions most directly related to reflective processing (understanding, reflection, and critical reflection), *r* = 0.451, *p* < 0.001. This association is consistent with convergent validity while remaining sufficiently below unity to indicate that objective text quality and self-reported reflective disposition are not redundant measures.

Manipulation checks. Post-task items assessed whether participants recognized the intended training and tool conditions. These items were retained as checks on assignment comprehension rather than as outcome measures. Their role is to confirm that the experimental conditions were psychologically distinguishable.

#### 3.1.4 Procedure and statistical analysis

All participants completed the pre-test case and submitted a written reflection report before any intervention. One week later, they received either the metacognitive or placebo training and then completed the post-test case under the assigned tool condition. The same task duration was used across conditions. Reflection reports were scored independently using the same rubric.

A sensitivity power analysis using G*Power (Faul et al., 2007) indicated that the obtained sample (*N* = 60) provided adequate power to detect the focal interaction effect. The primary analysis was a 2 × 2 ANCOVA predicting post-test written reflection quality from metacognitive training, tool condition, and their interaction while controlling for pre-test reflection quality. Before interpretation, homogeneity of regression slopes, homogeneity of variance, residual normality, and influence diagnostics were examined. Simple effects were estimated from the fitted model to interpret the interaction. Robustness checks included HC3 heteroskedasticity-robust inference, exclusion of observations exceeding the heuristic Cook’s D > 4/*N* threshold, and a per-protocol sensitivity analysis based on self-reported adherence to assigned tool use.

### 3.2 Results

#### 3.2.1 Baseline comparability, diagnostics, and manipulation checks

Pre-test written reflection quality did not differ significantly across the four groups, *F*(3, 56) = 1.11, *p* = 0.352. The serial number (1–60) was retained as the unique case key because two initials-based identifiers were duplicated in the source files.

ANCOVA diagnostics were acceptable. Homogeneity of regression slopes was supported, *F* = 1.50, *p* = 0.225; Levene’s test was non-significant, *F* = 0.92, *p* = 0.437; and residuals did not significantly depart from normality, Shapiro–Wilk *W* = 0.985, *p* = 0.668. The largest externally studentized residual was 2.66. 4 cases exceeded Cook’s D > 4/*N*; excluding them left the interaction significant, *F* = 10.41, *p* = 0.002. HC3 inference likewise retained the interaction (*p* = 0.004), and the per-protocol analysis (*N* = 53) produced the same conclusion, *F* = 11.34, *p* = 0.002.

#### 3.2.2 Primary ANCOVA: written reflection quality

After baseline adjustment, metacognitive training showed a significant main effect, *F*(1, 55) = 33.89, *p* < 0.001, *η*^2^*p* = 0.381. Tool condition also showed a significant main effect, *F*(1, 55) = 9.63, *p* = 0.003, *η*^2^*p* = 0.149. The predicted metacognition × tool interaction was significant, *F*(1, 55) = 10.91, *p* = 0.002, *η*^2^*p* = 0.165. The pre-test covariate was marginal, *F*(1, 55) = 3.76, *p* = 0.058.

Simple-effects analysis clarified the interaction. Under metacognitive training, AI-based Socratic scaffolding exceeded traditional tools by 0.56 model-estimated points, t(55) = 4.53, *p* < 0.001. Without metacognitive training, the tool contrast was negligible (Δ*M* = −0.03), t(55) = −0.23, *p* = 0.821. Metacognitive training itself was strongly beneficial in the AI condition (Δ*M* = 0.79, *p* < 0.001), whereas the corresponding difference under traditional tools was smaller and not significant (Δ*M* = 0.21, *p* = 0.101). This pattern supports H1, H1a, and H1b while also showing that the interaction is driven by the particularly strong performance of the metacognition + AI condition.

#### 3.2.3 Dimension-level pattern of written reflection

The interaction was strongest for depth/attribution and strategy generation/transfer, the dimensions most directly tied to higher-order reflective processing. The interaction was weaker for basic description and report structure. This differentiated pattern is consistent with the proposition that metacognitive preparation is especially consequential when the AI system is used for causal analysis, perspective coordination, and the generation of justified alternatives rather than for lower-level descriptive work.

#### 3.2.4 Secondary RPQ evidence

The 16-item RPQ showed acceptable-to-good reliability at both time points (overall *α* = 0.808 at pre-test and 0.878 at post-test). The four-dimensional exploratory structure was also interpretable. Post-test written reflection quality correlated positively with the combined understanding, reflection, and critical-reflection dimensions (*r* = 0.451, *p* < 0.001). These results provide secondary convergent support, but the small sample does not justify treating the RPQ factor analysis as a definitive validation study.

## 4 Study 2: process-level human-AI interaction analysis

### 4.1 Method

#### 4.1.1 Data source

Study 2 used the dialogue logs from the two conditions that interacted with the AI-based Socratic system in Study 1: Group A (metacognitive training + AI; *n* = 15) and Group B (placebo training + AI; *n* = 15). After excluding system errors, blank inputs, and non-task-related small talk, the log dataset comprised 487 user prompts, with 254 prompts from Group A and 233 from Group B. The total number of interactions did not differ significantly between groups, t(28) = 1.12, *p* > 0.05, reducing the likelihood that group differences in prompt composition merely reflected different interaction volume.

#### 4.1.2 Unit of analysis and coding scheme

Each user prompt served as the basic coding unit. Drawing on the revised Bloom’s Taxonomy (Anderson and Krathwohl, 2001) and the ICAP framework, prompts were classified into three cognitive levels. Category 1 (surface-level inquiry) included factual retrieval, definitional requests, and direct answer seeking. Category 2 (procedural inquiry) included application, explanation, and context-linked procedural questions. Category 3 (metacognitive-driven inquiry) included critical evaluation, hypothetical reasoning, multi-perspective exploration, and logical verification (Walsh and Sattes, 2015).

Two doctoral students in educational technology independently coded a randomly selected 20% of the prompts before coding the remaining data. Inter-rater agreement for the initial set was high (Cohen’s *κ* = 0.86). Disagreements in the full coding process were resolved by discussion. This analysis characterizes the distribution and participant-level prevalence of interaction behaviors; it is not a statistical mediation model linking prompt characteristics to Study 1 outcomes.

### 4.2 Results

#### 4.2.1 Distribution of prompt categories

The two groups displayed markedly different prompt profiles. In Group B, 54.5% of prompts were classified as lower-order inquiries and 12.2% as higher-order inquiries. In Group A, lower-order inquiries accounted for 21.3% and higher-order inquiries for 44.1%. Intermediate inquiries were similar across groups (34.6% vs. 33.3%). Thus, metacognitive training was associated with a shift away from direct answer seeking toward prompts that asked the AI system to compare explanations, evaluate reasoning, or explore alternatives.

#### 4.2.2 Participant-level comparison of higher-order inquiry

For each participant, the proportion of Category 3 prompts was calculated relative to that participant’s total prompts. The metacognitive-training group showed a substantially larger proportion of higher-order inquiries (*M* = 0.44, SD = 0.15) than the placebo-training group (*M* = 0.12, SD = 0.09), t(28) = 7.14, *p* < 0.001, *d* = 2.61. This finding supports H2: metacognitive preparation corresponded to a qualitatively different pattern of interaction with the same configured AI system.

The result should nevertheless be interpreted as process-level evidence. Study 2 did not test whether the proportion or linguistic properties of higher-order prompts statistically mediated the effect of training on post-test reflection quality. Moreover, because the primary Study 1 interaction was not significant in the reanalysis, the Study 2 pattern cannot be described as explaining a demonstrated moderation effect. It instead shows that metacognitive training changed how participants used the AI system, a behavioral difference that is theoretically relevant to reflective engagement and warrants direct mediation testing in a larger study.

#### 4.2.3 Illustrative interaction patterns

The qualitative contrast was also visible in interaction excerpts from the same teaching scenario. Placebo participants commonly asked for judgments, definitions, or ready-made improvement plans. Metacognitively trained participants more often framed competing explanations, proposed counterfactual alternatives, and asked the system to critique their own theoretical reasoning. These examples illustrate the coding categories and complement the quantitative comparison; they are not used as independent causal evidence.

#### 4.2.4 Summary

Study 2 indicates that metacognitive training can reorganize the form of human-AI dialogue even when the technological interface is held constant. Trained participants engaged the AI system with more evaluative and exploratory prompts rather than merely increasing the number of prompts. This pattern is consistent with the proposition that metacognitive strategies influence the process of AI-supported reflection. The current evidence stops short of establishing a mediational pathway from training through prompt quality to reflection outcomes.

## 5 General discussion

Across the two studies, the evidence points to a conditional relationship between metacognitive preparation and AI-supported reflective practice. Study 1 showed that both metacognitive strategy training and the configured AI-based Socratic environment were associated with higher written reflection quality, but the key finding was their interaction. The model-estimated advantage of AI-based Socratic scaffolding was substantial under metacognitive training and effectively absent under placebo training. Study 2, in turn, showed that trained AI users generated a markedly higher proportion of higher-order prompts. Together, these findings are consistent with the view that learner preparation shapes how the affordances of a dialogic AI system are used.

### 5.1 Interpreting the interaction effect

The interaction qualifies a simple technology-centered account. Access to a dialogic AI system did not yield a comparable improvement for participants without metacognitive preparation. Under metacognitive training, by contrast, the AI condition produced a model-estimated advantage of approximately 0.56 points over traditional digital tools. The result is consistent with self-regulated learning accounts in which planning, monitoring, and evaluation allow learners to use external support more selectively and strategically.

At the same time, the experiment does not isolate a generic AI effect. The AI condition contained Socratic questioning, adaptive dialogue, and feedback structure in addition to generative capability. The appropriate interpretation is therefore that metacognitive preparation strengthened performance within this configured AI-based Socratic environment. Whether the same interaction would occur with unrestricted chatbots, static prompt templates, or other AI systems remains an empirical question.

### 5.2 Process-level evidence from study 2

Study 2 adds behavioral detail to the outcome pattern. The metacognitive-training group generated substantially more higher-order prompts and fewer direct answer-seeking prompts. This difference suggests that training was expressed in the interaction itself: participants more often asked for counterarguments, causal explanations, alternative perspectives, and evaluation of their own reasoning.

Behavioral correspondence should not be equated with mediation. The present log analysis does not estimate an indirect effect linking training, prompt quality, and reflection outcome at the participant level. Accordingly, the dialogue findings are described as process-level evidence consistent with the proposed pathway rather than as proof that prompt behavior statistically mediated the Study 1 interaction.

### 5.3 Measurement and methodological implications

The measurement re-audit also clarifies the evidentiary base of the study. The reflection rubric shows strong inter-rater reliability and an explicit theoretical mapping, but those two features serve different purposes: ICC establishes scoring consistency, whereas construct validity depends on the conceptual adequacy of the dimensions and their relationship to external indicators. The moderate correlation between rubric-based post-test quality and RPQ reflective dimensions provides additional, though not definitive, convergence.

For the RPQ, separating the 16 intended scale items from two background questions materially improved the coherence of the measurement model. The acceptable reliability coefficients, KMO values, Bartlett tests, and four-component exploratory structure support its use as a secondary self-report measure in this sample. Because the sample is small for latent-variable validation, these psychometric results should not be presented as a definitive cross-cultural validation of the adapted scale.

### 5.4 Implications for teacher education and educational AI

For teacher educators, the results support pairing AI-supported reflection with explicit training in self-questioning, evidence evaluation, goal setting, and strategy monitoring. Tool access alone is not the same as productive use (Mollick, 2024). Programs can therefore place greater emphasis on preparing learners to interrogate AI output, compare alternatives, and justify decisions rather than simply teaching prompt syntax.

For program and policy design, implementation should specify the pedagogical role assigned to AI, the form of adaptive guidance, expectations for verification, and the learner preparation that accompanies tool access. The present evidence supports this principle within a configured Socratic reflection system; it does not justify a universal claim that any AI tool will produce the same effects.

For system developers, the findings motivate configurable forms of productive friction. A reflection system can require users to state an initial interpretation, request evidence for a claim, compare alternative explanations, or revisit an earlier assumption before receiving a complete answer. Whether these functions outperform simpler non-AI scaffolds should be tested through component or dismantling experiments.

### 5.5 Limitations and future research

Several limitations delimit the conclusions. First, the sample comprised 60 participants, only 15 per factorial cell. The observed interaction was robust in the present sensitivity analyses, but small cell sizes reduce precision and limit subgroup generalizability. Larger preregistered replications are needed.

Second, participants were pre-service teachers from a single Chinese university. Cultural norms surrounding teacher authority, reflection, and technology use may shape both training responses and AI interaction patterns. Cross-institutional and cross-cultural replication is necessary before broader generalization.

Third, the technological contrast included multiple bundled affordances. AI generation, adaptive questioning, dialogic interaction, and feedback were not separately manipulated. Future dismantling studies should distinguish these components.

Fourth, the intervention was short and the outcomes were measured immediately. The study cannot determine whether gains persist, transfer to authentic classroom practice, or change as AI experience accumulates. Longitudinal follow-up is therefore essential.

Fifth, the RPQ psychometric check was conducted in a small experimental sample. The revised scoring improves internal consistency and structural coherence, but larger samples are required for robust confirmatory factor analysis, measurement invariance, and cross-cultural validation.

Finally, the manuscript clarifies its novelty on the basis of evidence available in this study rather than attempting an unsupported comparison with the confidential submission referenced by Reviewer 2. Its distinctive contribution lies in experimentally separating metacognitive preparation from a configured AI-based Socratic condition and combining this design with process-level dialogue analysis.

## 6 Conclusion

This dual-study investigation examined whether metacognitive preparation changes the effectiveness and use of an AI-based Socratic reflection environment. Study 1 showed significant main effects of metacognitive training and tool condition together with a significant interaction. AI-based Socratic scaffolding was associated with a clear improvement in written reflection quality when participants had received metacognitive training, whereas no comparable tool advantage was observed under placebo training. Study 2 showed that metacognitively trained AI users generated a substantially larger proportion of higher-order prompts.

The evidence therefore supports a conditional but bounded conclusion. Metacognitive preparation appears to help pre-service teachers use a configured dialogic AI system in ways that support deeper short-term reflective performance. The study does not establish that generic AI access is inherently empowering, nor does the dialogue-log analysis constitute a statistical mediation test. The contribution lies in showing that learner regulation and interaction design should be considered jointly when evaluating AI-supported reflection.

For teacher education, the practical implication is to integrate AI-supported reflective tasks with explicit metacognitive preparation and process-oriented evaluation. Whether these benefits endure over time, generalize across cultures and tasks, or transfer to authentic teaching practice remains a question for larger longitudinal research.

## Data Availability

The raw data supporting the conclusions of this article will be made available by the authors, without undue reservation.

## References

[ref1] Almufarreh A. (2024). Determinants of students’ satisfaction with AI tools in education: a PLS-SEM-ANN approach. Sustainability 16:5354. doi: 10.3390/su16135354

[ref2] Anderson L. W. Krathwohl D. R. (Eds.). (2001). A Taxonomy for Learning, Teaching, and Assessing: A Revision of Bloom’s Taxonomy of Educational Objectives. New York: Longman.

[ref3] Ansari A. N. Ahmad S. Bhutta S. M. (2023). Mapping the global evidence around the use of ChatGPT in higher education: a systematic scoping review. Educ. Inf. Technol. 29, 11281–11321. doi: 10.1007/s10639-023-12223-4

[ref4] Azevedo R. Hadwin A. F. (2005). Scaffolding self-regulated learning and metacognition: implications for the design of computer-based scaffolds. Instr. Sci. 33, 367–379. doi: 10.1007/s11251-005-1272-9

[ref5] Baidoo-Anu D. Ansah L. O. (2023). Education in the era of generative artificial intelligence (AI): understanding the potential benefits of ChatGPT in promoting teaching and learning. J. AI 7, 52–62. doi: 10.61969/jai.1337500

[ref6] Bates T. Cobo C. Mariño O. Wheeler S. (2020). Can artificial intelligence transform higher education? Int. J. Educ. Technol. High. Educ. 17:42. doi: 10.1186/s41239-020-00218-x

[ref7] Bearman M. Ajjawi R. (2023). Learning to work with the black box: pedagogy for a world with artificial intelligence. Br. J. Educ. Technol. 54, 1160–1173. doi: 10.1111/bjet.13337

[ref8] Bearman M. Ryan J. Ajjawi R. (2023). Discourses of artificial intelligence in higher education: a critical literature review. High. Educ. 86, 369–385. doi: 10.1007/s10734-022-00937-2

[ref9] Bransford J. D. Brown A. L. Cocking M. R. (Eds.). (2000). How People Learn: Brain, Mind, Experience, and School. Washington, DC: National Academy Press.

[ref10] Chan C. K. Y. Hu W. (2023). Students’ voices on generative AI: perceptions, benefits, and challenges in higher education. Int. J. Educ. Technol. High. Educ. 20:43. doi: 10.1186/s41239-023-00411-8

[ref11] Chen R. (2022). Research on the assessment and cultivation strategies of reflective practice ability for teacher professional development [Doctoral Dissertation, Central China Normal University]. CNKI. doi: 10.27159/d.cnki.ghzsu.2022.004008 [in Chinese]

[ref12] Collins A. Brown J. S. Newman S. E. (1989). “Cognitive apprenticeship: teaching the crafts of reading, writing, and mathematics,” in Knowing, Learning, and Instruction: Essays in honor of Robert Glaser, ed. Resnick L. B. (Hillsdale: Lawrence Erlbaum Associates), 453–494.

[ref13] Crompton H. Burke D. (2023). Artificial intelligence in higher education: the state of the field. Int. J. Educ. Technol. High. Educ. 20:22. doi: 10.1186/s41239-023-00392-8

[ref14] Delcker J. Heil J. Ifenthaler D. Seufert S. Spirgi L. (2024). First-year students AI-competence as a predictor for intended and de facto use of AI-tools for supporting learning processes in higher education international journal of educational Technology in Higher. Education 21:18. doi: 10.1186/s41239-024-00452-7

[ref15] Desoete A. (2008). Multi-method assessment of metacognitive abilities in elementary school children: how you test is what you get. Metacogn. Learn. 3 3, 189–206. doi: 10.1007/s11409-008-9026-0

[ref16] Dewey J. (1938). Experience and Education. Kappa Delta Pi.

[ref17] Dimmitt C. McCormick C. B. (2012). “Metacognition in education,” in APA Educational Psychology Handbook: Vol. 1. Theories, Constructs, and Critical Issues, eds. Harris K. R. Graham S. Urdan T. McCormick C. B. Sinatra G. M. Sweller J. (Eds.), (Washington, DC: American Psychological Association), 157–187.

[ref18] Efklides A. (2008). Metacognition: defining its facets and levels of functioning in relation to self-regulation and co-regulation. Eur. Psychol. 13, 277–287. doi: 10.1027/1016-9040.13.4.277

[ref19] Faul F. Erdfelder E. Lang A. G. Buchner A. (2007). G*power 3: a flexible statistical power analysis program for the social, behavioral, and biomedical sciences. Behav. Res. Methods 39, 175–191. doi: 10.3758/BF03193146, 17695343

[ref20] Flavell J. H. (1979). Metacognition and cognitive monitoring: a new area of cognitive-developmental inquiry. Am. Psychol. 34, 906–911. doi: 10.1037/0003-066x.34.10.906

[ref21] Fošner A. (2024). University students’ attitudes and perceptions towards AI tools: implications for sustainable educational practices. Sustainability 16:8668. doi: 10.3390/su16198668

[ref22] Grájeda A. Burgos J. Córdova P. Sañés A. (2024). Assessing student-perceived impact of using artificial intelligence tools: construction of a synthetic index of application in higher education. Cogent Educ. 11:2287917. doi: 10.1080/2331186X.2023.2287917

[ref23] Hatton N. Smith D. (1995). Reflection in teacher education: towards definition and implementation. Teach. Teach. Educ. 11, 33–49. doi: 10.1016/0742-051x(94)00012-u

[ref24] Kasnakoglu T. (2023). What do ChatGPT’s capabilities imply about the capabilities required for education? J. Educ. Technol. Soc. 26, 1–14.

[ref25] Kember D. Leung D. Y. P. Jones A. Loke A. Y. McKay J. Sinclair K. . (2000). Development of a questionnaire to measure the level of reflective thinking. Assess. Eval. High. Educ. 25, 381–391.

[ref26] Kinsella E. A. (2007). Technical rationality in Schön’s reflective practice: dichotomous or non-dualistic epistemological position. Nurs. Philos. 8, 102–113. doi: 10.1111/j.1466-769X.2007.00304.x 17374071

[ref27] Korthagen F. A. J. Kessels J. (1999). Linking practice and theory: the pedagogy of realistic teacher education. Br. J. Educ. Stud. 25, 381–395. doi: 10.1080/713611442

[ref28] Kramarski B. Michalsky T. (2010). Preparing preservice teachers for self-regulated learning in the context of technological pedagogical content knowledge. Learn. Instr. 20, 434–447. doi: 10.1016/j.learninstruc.2009.05.003

[ref29] Lodge J. M. Howard S. K. Bearman M. Dawson P. (2024). Assessment reform for the age of artificial intelligence. Tert. Educ. Manag. 30, 1–15.

[ref30] Ma Y. Zheng X. Zhao R. Liu H. (2022). The occurrence mechanism and multimodal data evaluation of deep learning. Distance Educ. J. 40, 50–60. doi: 10.15881/j.cnki.cn33-1304/g4.2022.01.003 [in Chinese]

[ref31] Mollick E. (2024). Co-Intelligence: Living and Working with AI. New York: Portfolio.

[ref32] Moon J. A. (2013). A Handbook of Reflective and Experiential Learning: Theory and Practice. London: Routledge.

[ref33] Norman D. A. (1988). The Psychology of Everyday Things. New York: Basic Books.

[ref34] Ohtani K. Hisasaka T. (2018). Beyond intelligence: a meta-analytic review of the relationship among metacognition, intelligence, and academic performance. Metacogn. Learn. 13, 179–212. doi: 10.1007/s11409-018-9183-8

[ref35] Overholser J. C. (1997). Socratic psychotherapy. Psychother. Patient 10, 83–97.

[ref36] Reiser B. J. Tabak I. (2014). “Scaffolding,” in The Cambridge Handbook of the Learning Sciences, ed. Sawyer R. K.. 2nd ed (New York: Cambridge University Press), 44–62.

[ref37] Rolfe G. Freshwater D. Jasper M. (2001). Reflective Practice for Nurses: A Practical guide. 2nd Edn. Basingstoke: Palgrave.

[ref38] Schön D. A. (1983). The Reflective Practitioner: How Professionals Think in Action. New York: Basic Books.

[ref39] Schön D. A. (1987). Educating the Reflective Practitioner: Toward a new design for Teaching and Learning in the Professions. Jossey-Bass.

[ref40] Schraw G. (1998). Promoting general metacognitive awareness. Instr. Sci. 26, 113–125.

[ref41] Schraw G. Dennison R. S. (1994). Assessing metacognitive awareness. Contemp. Educ. Psychol. 19, 460–475. doi: 10.1006/ceps.1994.1033

[ref42] Schraw G. Moshman D. (1995). Metacognitive theories. Educ. Psychol. Rev. 7, 351–371. doi: 10.1007/bf02212307

[ref9003] Shearer R. L. Yu J. Peng X. (2021). Cognitive load and working memory: a system view of measurement. Learning: Research and Practice 7, 54–69. doi: 10.1080/23735082.2020.1830150

[ref43] Sparks-Langer G. M. Simmons J. M. Pasch M. Colton A. Starko A. (1990). Reflective pedagogical thinking: how can we promote it and measure it? J. Teach. Educ. 41, 23–32. doi: 10.1177/002248719004100504

[ref44] Van Manen M. (1977). Linking ways of knowing with ways of being practical. Curric. Inq. 6, 205–228. doi: 10.1080/03626784.1977.11075533

[ref45] Vygotsky L. S. (1978). Mind in Society: The Development of Higher Psychological Processes. Cambridge: Harvard University Press.

[ref46] Walsh K. Sattes B. (2015). Quality Questioning: Research-based Practice to Engage every Learner. Thousand Oaks: Corwin Press.

[ref47] Wang S. (2024). Design and application of reflective practice activities for practical knowledge cultivation in educational practicum [Master’s thesis, Northeast Normal University]. CNKI. [in Chinese]

[ref48] Wang B. Dou Q. (2013). Correlation study between reflective learning ability and academic achievement. J. Manag. 21, 84–86. [in Chinese]

[ref49] Ward J. R. McCotter S. S. (2004). Reflection as a visible outcome for preservice teachers. Teach. Teach. Educ. 20, 243–257. doi: 10.1016/j.tate.2004.02.004

[ref50] Wei G. (2020). The Generation of Teachers’ Practical Knowledge. Beijing: Educational Science Publishing House [in Chinese].

[ref51] York-Barr J. Sommers W. A. Ghere G. S. Montie J. (Eds.). (2005). Reflective Practice to Improve Schools: An action guide for Educators. Thousand Oaks: Corwin Press.

[ref9001] Zimmerman B. J. (2002). Becoming a self-regulated learner: An overview. Theory Into Practice, 41, 64–70.

