# Supplementary material for: Metacognitive training and AI-based Socratic scaffolding in pre-service teachers’ reflective practice: a dual-study experimental design
Source: Front Psychol. 2026 Oct 13;17:1960962. doi: 10.3389/fpsyg.2026.1960962 (PMC13710275; doi:10.3389/fpsyg.2026.1960962)
Supplement: Supplementary file 1 [file Data_Sheet_1.pdf]

Supplementary Material

1 Appendix A

Metacognitive Strategy Training Material: Deepening Teaching Reflection Using the P.O.S.T. Framework

Target Audience: All participants in the metacognitive intervention group (including both the AI tool group and the traditional tool group).

Duration: 20 minutes.

Objective: To enable participants to master the P.O.S.T. metacognitive framework and consciously apply it to guide their subsequent teaching reflection tasks. Part 1: Explanation—What is the P.O.S.T. Reflection Framework? (5 minutes)

(Trainer's Opening) "Hello everyone, welcome to this training. In our daily learning, we all engage in reflection and use various tools to search for information. However, a critical question remains: How can we reflect more 'smartly' and use tools more efficiently, rather than being overwhelmed by massive amounts of information? Today, I will introduce a simple yet powerful cognitive framework—the P.O.S.T. framework. It can help transform our teaching reflection from a random act into a strategic, in-depth process."

(PPT/Key Points)

P.O.S.T. is a structured reflection process representing four key steps:

P - Purpose

Core Question: "What specific problem do I actually want to solve?"

Explanation: Before starting to reflect or use any tools, pause for a minute. Instead of merely thinking, "I need to write a reflection report," define a more specific and valuable goal. For example, shift from "reflecting on a lesson" to "I want to find at least three methods to improve the engagement of students sitting in the back row." A clear purpose serves as the "compass" for your reflection journey.

O - Outline

Core Question: "To achieve my purpose, what key questions do I need to ask?"

Explanation: This is akin to formulating a "battle plan." Based on your purpose, break down the main problem into a series of specific, actionable sub-questions. This outline will directly guide how you prompt the AI or what keywords you enter into a search engine. For instance, based on the previous purpose, your outline might be: (1) What are the reasons for low engagement among back-

row students? (2) What teaching strategies can enhance classroom engagement? (3) Which strategies are particularly suitable for my subject (e.g., History/Mathematics)?

S - Scrutinize

Core Question: “What is the quality of the information provided by the tool? How should I evaluate and filter it?”

Explanation: This is the most crucial step, marking the divide between “passive reception” and “active thinking.” When a tool (whether AI or a search engine) provides an answer, never adopt it blindly. You must scrutinize it like a detective:

Is it relevant? Is this truly what I am looking for?

Is it reliable? What is the source of this perspective? Is there theoretical support?

Is it applicable? Is this method genuinely feasible in my specific class (e.g., 30 students/preschool)?

Is there a better alternative? Is this answer too superficial? Can I ask follow-up questions, or search from a different angle?

T - Transform

Core Question: “How do I transform this valuable information into my own specific, actionable plan?”

Explanation: Information itself has no value until you transform and apply it. This step requires you to integrate the high-quality information filtered from the tools with your own teaching context to form a context-specific improvement plan. For example: “Great, I’ve decided to adopt the ‘random calling + group discussion’ strategy. The specific steps are: First, prepare a random grouping list before class; Second, randomly ask a back-row group to answer when explaining key points; Third…”

Part 2: Example—Applying the P.O.S.T. Framework to Analyze the “Dinosaur Castle” Incident (10 minutes)

(Trainer’s Guidance) “Now, let’s use the P.O.S.T. framework we just learned to re-analyze the ‘Dinosaur Castle’ incident that you all encountered in the pre-test task. Let’s observe the thinking process of a teacher who applies metacognitive strategies.”

(PPT/Key Points)

Scenario Review: The teacher became angry because Xiaoming deviated from the teaching objective and took away his “castle,” causing Xiaoming to have an emotional breakdown.

A Teacher’s Thinking Process Using the P.O.S.T. Framework (General Case):

P (Purpose):

Surface Purpose: I need to reflect on this incident so I don’t make the same mistake next time.

Deep Purpose (Guided by P.O.S.T.): My goal is to find a balance between maintaining classroom order and protecting/guiding children’s creativity. I need specific strategies to handle similar “creative derailment” behaviors in the future. O (Outline): “To achieve this purpose, I need external tools to help me think. My question outline is:”

“Analyze the underlying child psychology reasons when a child’s personal interest conflicts with the collective teaching objective.”

“Provide 3-5 specific positive discipline strategies for handling such conflicts in the classroom.”

“Are there instructional design methods that can proactively integrate children’s interests into the curriculum to prevent such conflicts?”

S (Scrutinize):

“I found several suggestions through the tools, such as ‘setting clear rules,’

‘giving choices,’ and ‘integrated curriculum design.’”

My Scrutiny Process: “‘Setting rules’ is important, but in this case, it feels like hindsight. ‘Giving choices’ is an excellent point! For example, I could have said, ‘Do you want to spend 5 minutes building the castle first, and then 10 minutes molding the dinosaur; or mold the dinosaur first, and continue building the castle after class?’ The concept of ‘integrated curriculum design’ is a bit broad, but it inspired me. Perhaps I could say at the beginning of the craft class, ‘Everyone can build a home for your animal.’ This way, Xiaoming’s behavior would be within the rules. This finding is highly valuable!”

T (Transform): “Based on my scrutiny, I have formulated a new action plan for myself:” Emotional First-Aid Card: Next time I encounter a similar situation, take a deep breath first, and remind myself that the primary task is “connection” rather than “correction.”

Flexible Instructions: When assigning tasks, use more flexible language to leave room for creativity. For example, change “mold an animal” to “create an artwork about an animal.”

Golden Dialogue Script: I will say: ‘Wow, are you building a castle? That’s so cool! Can you tell me what your T-Rex will do in the castle? … Our task today is animals, how do you think you can make your castle and T-Rex complete this task together?’"

(Trainer’s Summary)

“Do you see? Through the P.O.S.T. framework, this teacher’s reflection is no longer a simple ‘I was wrong,’ but has formed a complete, analyzable, improvable, and actionable closed loop.”

Part 3: Practice and Consolidation (5 minutes)

(Trainer’s Task Assignment)

"To help you better master this framework, let's do a quick thinking exercise."

Scenario: "In your math class, a student named Xiaoli always finishes the exercises very quickly, then gets bored and starts tapping her pencil on the desk or talking to her deskmate, distracting other students."

Task: "Now, please take out a piece of paper and spend 3 minutes writing down your P.O.S.T. thinking outline for this scenario. You don't need to write a long essay; just write down:"

Your P (Purpose). Your O (At least 2 questions you want to ask the tool).

(After 2-3 minutes, Trainer Guides Sharing)

"Alright, time is up. Would anyone like to share your 'Purpose' and 'Question Outline'? Let's hear them."

(Invite 1-2 participants to share and provide positive feedback, e.g., "That purpose is very specific!" "Your second question has great depth and can guide you to a more fundamental solution.")

(Trainer's Conclusion)

"Excellent! You have all grasped the essence of the P.O.S.T. framework. In the upcoming T2 task, regardless of whether you are assigned to use AI tools or traditional search engines, please make sure to consciously use the P.O.S.T. framework to guide your entire reflection process. It will be a powerful weapon for deepening your thinking and improving the quality of your reflection. That concludes the training. Thank you all!"

2 Appendix B

Pre-test: T1

[Instructions for Participants]

Hello! Welcome to this study.

[Task Context]

Please carefully read the teaching scenario case regarding the "Dinosaur Castle" incident below. Now, imagine you are Teacher Li in the case. At the end of the workday, you need to review and reflect on this incident. Material Name: Scenario Case: The "Dinosaur Castle" Incident Scenario Description: Teacher Li is leading a group-based clay art class with the theme "My Favorite Animal." She distributes modeling clay to each group and asks the children to work together to sculpt their favorite animals.

Most children are enthusiastically discussing and sculpting, but Teacher Li notices that Xiaoming in Group 2 is not sculpting an animal. He has taken a large amount of brown clay and is focused on building a complex "castle." Teacher Li walks over and gently reminds him, "Xiaoming, our task

today is to sculpt small animals." Xiaoming replies without looking up, "I know, I'm building a home for my T-Rex. I'll put the T-Rex in when it's done."

Teacher Li feels that while Xiaoming's idea is creative, it deviates from the main objective of the lesson and might affect the progress of his group members. She persuades him again, "The castle is great, but can we finish the animal first? Otherwise, we might run out of time." Xiaoming is somewhat reluctant but puts down the "castle" and starts sculpting the T-Rex.

A few minutes later, Teacher Li finds Xiaoming secretly building his "castle"again, while his T-Rex is only roughly shaped. A girl in the same group complains to Teacher Li, "Teacher, Xiaoming isn't cooperating with us; he just keeps playing with the clay!" At this point, Teacher Li feels somewhat angry. She walks up to Xiaoming, directly takes away the "castle" he built, and says in a stern tone, "Please finish your animal immediately and stop doing irrelevant things!"

Xiaoming's eyes instantly turn red. He pushes the clay away, lies on the desk, and refuses to do anything else.

[Task Requirements]

Please independently write a scenario analysis report. Your report should include in-depth reflections on at least the following four questions: Event Review and Problem Diagnosis: Briefly describe the "Dinosaur Castle" incident. What do you think is the core conflict in this event? Multi-perspective Attribution Analysis: From the child's perspective, why do you think Xiaoming insisted on building the "castle" first? What thoughts and needs might underlie his behavior? From your perspective (Teacher Li), what was your goal at that moment? How did your emotions and decision-making evolve step by step?

Self-Critique and Behavioral Evaluation: Reviewing your three interventions throughout the incident, which actions do you consider effective and which were inappropriate? Why? What educational philosophies or teaching beliefs (e.g., regarding classroom discipline, teaching objectives, children's creativity) might your handling of the situation reflect?

Improvement and Enhancement Strategies: If you could go back in time, what different and more effective communication or guidance strategies might you adopt when you first discovered Xiaoming building the "castle"? Please describe at least two specific approaches and explain your rationale.

[Important Notes]

Please rely entirely on your own pedagogical knowledge and reasoning to complete this report.

During this process, the use of any external auxiliary tools, such as search engines, AI chat tools (e.g., ChatGPT), or discussions with others, is strictly prohibited.

Time Limit: The total time for writing the report must not exceed 30 minutes.

Word Count Requirement: The report must be no less than 400 words (electronic version acceptable).

Post-test: T2 [Instructions for Participants]

Hello! We are now entering the second phase of the study.

[Task Context]

Please carefully read the teaching scenario case regarding the “Magic Seed” incident below. Now, imagine you are Teacher Wang in the case. At the end of the workday, you need to review and reflect on this incident.

Material Name: Scenario Case: The “Magic Seed” Incident

Scenario Description:

Teacher Wang is organizing a science observation class with the theme “Seed Germination.” She gives each child a small flowerpot, some soil, and a few mung bean seeds. The objective is to guide the children to continuously observe and record the seed growth process through hands-on planting.

After the activity begins, the children earnestly fill their pots with soil. However, Teacher Wang notices that Lele takes out a small bottle of juice and a small bag of sparkling colored glitter from her backpack. Teacher Wang walks over and asks

curiously, “Lele, what are you going to do with those?” Lele whispers excitedly, “Teacher Wang, this is a magic seed. I’m going to water it with ‘magic potion’ and sprinkle ‘fairy dust’ on it, and it will grow into a rainbow tree!”

Teacher Wang smiles and acknowledges her imagination, but then guides her, “That’s a very creative idea! But this is a science class, and seeds need water, soil, and sunlight to sprout.” Lele looks a bit disappointed but nods and puts the juice and glitter away.

A while later, when the children are watering their seeds, a child in the same group raises his hand and says, “Teacher, Lele poured her juice into the flowerpot!” Teacher Wang walks over and finds that the soil in Lele’s pot is soaking wet, smells sweet, and has glitter sprinkled on it. Teacher Wang worries that Lele’s “incorrect operation” will mislead other children’s understanding of scientific knowledge and feels that her instructions were ignored. She directly takes away Lele’s flowerpot and says to her, “Lele, the seed won’t sprout if you do this. For this class, just watch how the other students do it.”

Lele stares at the confiscated flowerpot, freezes for a few seconds, then lies on the desk and begins to cry softly.

[Task Requirements]

Please must use the auxiliary tools provided in front of you to help complete this report.

(Instructions for AI Tool Group): Please use the AI dialogue tool open on your desktop. By asking questions, obtaining suggestions, and seeking different perspectives, use it to help you analyze the case, deepen your reflection, and write a high-quality analysis report.

(Instructions for Traditional Tool Group): Please use the web browser open on your desktop. By using search engines to find relevant educational theories, teaching strategies, and expert opinions, use it to help you analyze the case, deepen your reflection, and write a high-quality analysis report.

Your report should include in-depth reflections on at least the following four questions:

Event Review and Problem Diagnosis: Briefly describe the "Magic Seed" incident and diagnose the core conflict in the event.

Multi-perspective Attribution Analysis: From the child's perspective, analyze the thoughts and needs underlying Lele's behavior. From your perspective (Teacher Wang), analyze your goals, emotions, and decision-making changes at that moment. Self-Critique and Behavioral Evaluation: Review your interventions in the incident and evaluate their pros and cons. What educational philosophies or teaching beliefs might your handling of the situation reflect? Improvement and Enhancement Strategies: If you could do it over again, what different and more effective communication or guidance strategies would you adopt? Please describe at least two specific approaches and explain your rationale.

[Important Notes]

Time Limit: The total time for writing the report is 40 minutes. (Note: Slightly longer than the pre-test to allow extra time for interacting with the tools).

Word Count Requirement: The report must be no less than 400 words.

3 Appendix C

Quality Scoring Rubric for Pre-service Teachers' Teaching Reflection Reports

Research Project: Enhancing the Empowering Effect of AI on Pre-service Teachers' Reflective Practice through Metacognitive Intervention: A Dual-Experiment Study

Report ID: _______________

Rater ID: _______________

Date of Scoring: _______________

Instructions for Raters

Blind Scoring Principle: You should remain unaware of the experimental group to which the report belongs. Please conduct an independent and objective evaluation based solely on the report text.

Focus on Criteria: Please score strictly according to the five dimensions and their corresponding rating descriptions defined in the table below. Avoid letting personal preferences or familiarity with the report topic influence your judgment.

Holistic Scoring: Please read through the entire report first to form a general impression, and then score each item individually.

Integer Scoring: Please assign an integer score from 1 to 5 for each dimension.

Remarks: If you encounter situations that are difficult to define or particularly noteworthy, please briefly

explain them in the “Overall Comments & Notes” section.

Scoring Rubric

Dimension	Weight	Rating Scale and Description(1-5 points)	Score
1.Description of Teaching Facts and Evidence Awareness (Corresponding to “O” in P.O.S.T.)	20%	5 (Excellent): Objective, specific, and detailed description of teaching events. Able to cite multiple specific student behaviors, language, or artifacts as evidence to support observations, rather than relying on intuition.<br>4 (Good): Relatively objective and specific description; able to cite at least one specific student behavior or language as evidence, but not sufficient or in-depth.<br>3 (Fair): Primarily a chronological narrative of the teaching process; mentions student reactions but lacks specific evidence, with considerable subjectivity.<br>2 (Poor): Vague, generalized, or overly simplified description; difficult to understand the actual classroom situation.<br>1 (Missing): Fails to describe relevant teaching events.	
2. Depth of Reflection and Attribution Analysis (Corresponding to “S” in P.O.S.T.)	30%	5 (Excellent): Deeply explores the root causes of the event; able to conduct systematic attribution from multiple dimensions (e.g., own teaching behavior, student characteristics, instructional design, environmental factors) rather than single attribution or shifting blame.<br>4 (Good): Analyzes the causes of the event, but may lack depth, or primarily focuses on one or two dimensions of attribution.<br>3 (Fair): Identifies successes or failures, but attribution is superficial (e.g., “students were not interested,” “I was not well-prepared”), lacking deep exploration.<br>2 (Poor): Merely states whether the phenomenon was good or bad without conducting attribution analysis.<br>1 (Missing): No evaluation or analysis of the event.	
3.Theoretical Connection and Perspective-Taking (Corresponding to “P” and “S” in P.O.S.T.)	20%	5 (Excellent): Clearly connects teaching practice with at least one relevant educational theory (e.g., constructivism, cognitive load) and effectively engages in perspective-taking (e.g., examining own teaching from the perspective of students, mentor teachers, or subject experts).<br>4 (Good): Attempts to connect with educational theories or engage in perspective-taking, but the connection is not precise or the perspective shift is incomplete.<br>3 (Fair): Vaguely mentions certain educational concepts or others’ perspectives, but does not elaborate on the analysis, or remains at the level of common sense.<br>2 (Poor): Completely confined to one’s own perspective; shows no awareness of theoretical thinking or empathy/perspective-taking.<br>1 (Missing): No mention of any theory or others’ perspectives.	
4.Generation and	20%	5 (Excellent): Based on reflective analysis, proposes	

Transfer of Improvement Strategies (Corresponding to "T" in P.O.S.T.)		specific, actionable, and well-founded improvement strategies, and further considers how these strategies or principles derived from reflection can be transferred to other future teaching contexts.<br>4 (Good): Proposes specific improvement strategies, but the rationale is insufficient, or fails to clearly articulate their transferability.<br>3 (Fair): Proposes directions for improvement, but strategies are vague (e.g., "be more patient next time," "prepare more"), lacking operability.<br>2 (Poor): Aware of the need for improvement, but fails to propose any improvement strategies.<br>1 (Missing): No mention of any ideas regarding future improvement.	
5. Structure and Clarity of the Report	10%	5 (Excellent): Clear structure, strong logic (e.g., reflects the logic of the P.O.S.T. framework); language is professional, accurate, and fluent.<br>4 (Good): Generally clear structure, coherent logic, and relatively fluent language.<br>3 (Fair): Somewhat loose structure, unclear logic in some paragraphs, and relatively colloquial language.<br>2 (Poor): Disorganized structure, lack of coherence, difficult to understand.<br>1 (Missing): Incomplete report format or severe issues in language expression.	
Total Score	100%	Calculation Formula: (Dimension 1 Score * 0.2) + (Dimension 2 Score * 0.3) + (Dimension 3 Score * 0.2) + (Dimension 4 Score * 0.2) + (Dimension 5 Score * 0.1)	Final Score: _____

4 Appendix D

Coding Scheme for Human-AI Dialogue Logs

Research Project: Enhancing the Empowering Effect of AI on Pre-service Teachers' Reflective Practice through Metacognitive Intervention: A Dual-Experiment Study

1. Coding Instructions

Unit of Analysis : The primary unit of analysis is a "Dialogue Turn", defined as one prompt submitted by the pre-service teacher and the corresponding response generated by the AI.

Coding Rule: A single dialogue turn can be assigned multiple codes across different main categories (e.g., a prompt can simultaneously exhibit "Contextualization" and "Attribution Analysis"), but codes within the same sub-category are mutually exclusive.

Reliability : Two independent coders will code 20% of the dialogue logs to calculate Cohen's Kappa. Discrepancies will be resolved through discussion.

2. Coding Rubric

Main Category	Code	Description	Example from Logs
A.Prompting Strategies Focuses on how users interact with AI.	A1.Direct Questioning	Asking straightforward questions without providing specific context or constraints.	"How should I handle a student who doesn't follow instructions?"
	A2.Contextualization	Providing specific background information, student details, or teaching scenarios before asking.	"In a science class about seeds, a 5-year-old girl used juice instead of water. How should I guide her?"
	A3. Role-Playing	Assigning a specific persona or role to the AI (e.g., expert teacher, student).	"Act as an experienced kindergarten teacher and evaluate my handling of the 'Magic Seed' incident."
	A4.Iterative Refinement	Modifying, narrowing down, or expanding a previous prompt based on the AI's prior response.	"Your previous answer was too general. Please give me two specific strategies based on constructivism."
B.Reflective Dimensions Based on the P.O.S.T. framework.	B1. Fact Observation (O)	Requesting AI to help summarize, extract, or clarify the objective facts of the teaching incident.	"Help me summarize the timeline of the 'Dinosaur Castle' event based on the text."
	B2. Sense-making & Attribution (S)	Exploring the underlying causes of student behaviors or teacher emotions.	"Why do you think Lele insisted on using 'magic potion'? What psychological need does this reflect?"

	B3. Perspective-Taking (P)	Analyzing the event from alternative viewpoints (e.g., the child's, parents', or theorists' perspectives).	"If you were Xiaoming, how would you feel when the teacher took away the castle?"
	B4. Strategy Transfer (T)	Generating actionable solutions or discussing how to apply learned principles to future scenarios.	"Based on this reflection, what specific steps can I take next time to protect creativity while maintaining discipline?"
C. Metacognitive Regulation Focuses on self-monitoring during AI use.	C1. Evaluating AI Output	Critically assessing the AI's response for accuracy, relevance, or bias, rather than accepting it blindly.	"I don't completely agree with your second point. In a real classroom, taking away the toy directly might cause a meltdown."
	C2. Clarifying Intentions	Explicitly stating one's own learning goals or cognitive needs to guide the AI.	"I am not looking for a direct answer. I want you to ask me guiding questions so I can reflect on my own."
	C3. Self-Correction	Recognizing flaws in one's own previous thinking or prompts and adjusting them.	"Wait, I realized my previous prompt was biased against the student. Let's re-analyze this objectively."

3. Data Extraction and Analysis

Frequency Analysis: Calculate the frequency and proportion of each code across the Metacognitive Intervention Group (Experimental) and the Control Group.

Sequential Analysis: Conduct lag-sequential analysis (LSA) to identify significant behavioral transitions (e.g., does C1. Evaluating AI Output frequently lead to A4. Iterative Refinement?).

Correlation with Report Quality: Correlate the frequency of high-level codes (e.g., B3, B4, C1, C2) with the final scores of the teaching reflection reports (Appendix III) to determine the effectiveness of specific human-AI interaction patterns.
